# Dietary astragalin confers protection against lipopolysaccharide-induced intestinal mucosal barrier damage through mitigating inflammation and modulating intestinal microbiota

**DOI:** 10.3389/fnut.2024.1481203

**Published:** 2024-10-02

**Authors:** Enhui Tang, Huan Lin, Yihao Yang, Jiawen Xu, Baiwen Lin, Yang Yang, Zijian Huang, Xinlan Wu

**Affiliations:** School of Public Health, Guangzhou Medical University, Guangzhou, China

**Keywords:** astragalin, intestinal microbiota, intestinal mucosal barrier damage, tight junction, inflammation

## Abstract

**Introduction:**

The intestinal mucosal barrier (IMB) damage is intricately linked with the onset of numerous intestinal diseases. Astragalin (AS), a flavonoid present in numerous edible plants, exhibits notable antioxidant and anti-inflammatory properties, demonstrating a promising impact on certain intestinal ailments. In this study, our objective was to investigate the protective effects of AS and elucidate the underlying mechanisms by which it mitigates lipopolysaccharide (LPS)-induced damage to the IMB in mice.

**Methods:**

During the experimental period, mice were subjected to a 7-day regimen of AS treatment, followed by LPS injection to induce IMB damage. Subsequently, a comprehensive evaluation of relevant biological indicators was conducted, including intestinal pathological analysis, serum inflammatory factors, intestinal tight junction proteins, and intestinal microbiota composition.

**Results:**

Our results suggested that AS treatment significantly bolstered IMB function. This was evidenced by the enhanced morphology of the small intestine and the elevated expression of tight junction proteins, including ZO-1 and Claudin-1, in addition to increased levels of MUC2 mucin. Moreover, the administration of AS demonstrated a mitigating effect on intestinal inflammation, as indicated by the reduced plasma concentrations of pro-inflammatory cytokines such as IL-6, IL-1β, and TNF-α. Furthermore, AS treatment exerted a positive influence on the composition of the gut microbiota, primarily by augmenting the relative abundance of beneficial bacteria (including *Lachnospiracea* and *Lactobacillus murinus*), while simultaneously reducing the prevalence of the harmful bacterium Mucispirillum schaedleri.

**Conclusion:**

AS mitigates LPS-induced IMB damage via mitigating inflammation and modulating intestinal microbiota.

## 1 Introduction

The intestinal mucosal barrier (IMB) serves as the boundary between the external environment and the body’s internal milieu, primarily comprising intestinal epithelial cells (IECs), gut microbiota, tight junctions (TJs), a mucus layer, and immune cells (1, 2). This barrier is essential for maintaining human health by preventing the entry of bacteria, antigens, and harmful substances from the intestinal lumen, while facilitating nutrient absorption (3). Several factors, such as the Western diet, non-steroidal anti-inflammatory drugs, food allergens, and lipopolysaccharides (LPS), can compromise the integrity of the IMB (4). The breakdown of this barrier often accompanies intestinal disorders, including irritable bowel syndrome (IBS), Crohn’s disease, and ulcerative colitis. Over the past few years, the occurrence of intestinal diseases has steadily increased worldwide, leading to a significant burden on the medical and healthcare systems (5). For example, IBS is known to affect approximately 11.2% of the world’s population (6). Previously, IBS was primarily diagnosed in the populations of certain Western countries, but now it is also occurring in some newly industrialized countries (7). For instance, India has the highest incidence rate in Asia (8). Therefore, it is important to explore preventive or therapeutic methods to protect the IMB.

The human intestinal microbiota forms a highly diverse ecosystem, comprising trillions of microorganisms crucial for regulating and preserving the function of the IMB (9, 10). This microbiota is capable of producing short-chain fatty acids (SCFAs), through the breakdown of certain indigestible carbohydrates like dietary cellulose and resistant starch (11). SCFAs are crucial energy substances for IECs and can maintain intestinal homeostasis through their anti-inflammatory effects (12). Moreover, intestinal microbiota can maintain intestinal health by secreting antimicrobial peptides to defend against invading pathogens and harmful microorganisms (13). Hence, preserving a balanced and healthy gut microbiota is instrumental in ensuring the integrity of the IMB.

Nowadays, drug therapy is the main means to prevent and treat intestinal barrier injury. However, it often achieves unsatisfactory results and may cause side effects (14, 15). In this regard, natural plant compounds have attracted much attention due to their efficacy and minimal side effects. Astragalin (AS), a naturally occurring flavonoid compound present in a variety of edible plants such as green tea seeds, mulberry, and *Moringa oleifera* leaves, possesses diverse biological activities and significant medicinal value, including anti-cancer, antioxidant, anti-ulcer, and anti-inflammatory properties (16, 17). Intestinal barrier injury can be caused by various factors. Among them, LPS, a critical component of the cell walls of Gram-negative bacteria, is particularly known for inducing inflammatory responses and compromising the IMB (18). Notably, studies have demonstrated not only the beneficial effects of AS in ameliorating LPS-induced inflammatory responses in both microglia and kidneys but also its efficacy in alleviating acute experimental colitis in mice, thereby suggesting its potential for protecting the IMB (19–21). Here, we utilized LPS to induce IMB damage in mice and investigated the protective effects of AS treatment on IMB integrity, with a particular focus on its impact on the intestinal flora.

## 2 Materials and methods

### 2.1 Animal experiments

Forty male C57BL/6 mice (6 weeks old) were obtained from the Guangdong Medical Laboratory Animal Center [production license number: SCXK (Guangdong) 2022–0002, Guangdong, China]. The mice were housed in a meticulously controlled environment [temperature: 23 ± 3°C, relative humidity (RH): 40–70%], and a 12-h light–dark cycle. The mice were kept in a total of 8 cages, each housing 5 mice. Throughout the study, they had unrestricted access to standard food and water.

**Figure 1:**
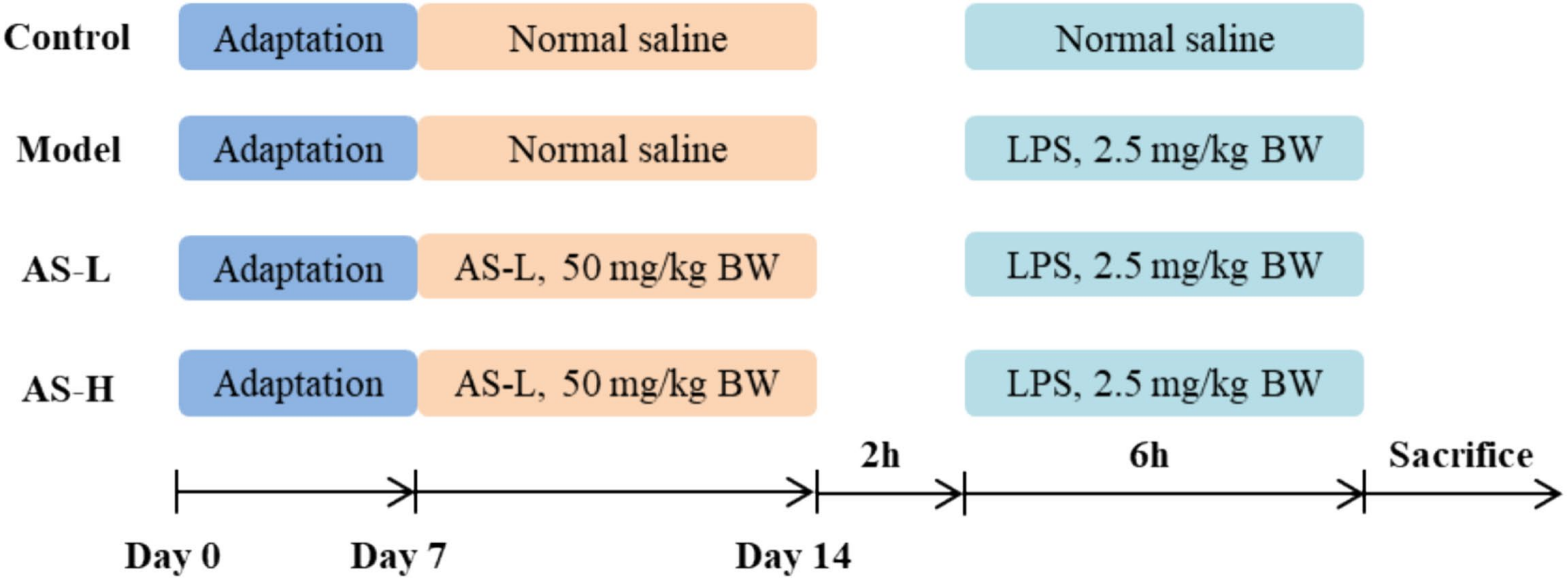
Scheme of the animal experimental design.

The overarching experimental protocols and procedures for conducting studies with mice are comprehensively illustrated in Figure 1. After a 7-day acclimatization period, the mice were assigned into four distinct groups in a random manner: Model, Control, low dose of AS (AS-L, 50 mg/kg BW), and high dose of AS (AS-H, 100 mg/kg BW). AS was dissolved in normal saline (NS) with a purity of ≥98% (Vicki Biotechnology, Sichuan, China). Mice in the Model and Control groups were administered a daily intragastric volume (0.1 mL/10 g BW) of NS. Mice in the AS-L and AS-H groups underwent daily intragastric dosing with AS at the corresponding doses. On the 7th day, 2 h after intragastric administration, mice in the AS-treated groups and Model group were received intraperitoneal injections of LPS (dissolved in NS, 2.5 mg/kg BW, O55: B5, #L2880, Sigma-Aldrich, Saint Louis, MO, United States). Meanwhile, the mice in the Control group were given an equivalent volume of NS injection (0.1 mL/10 g BW). Six hours post-LPS injection, mice were euthanized using CO_2_ gas anesthesia. Blood was collected from the retro-orbital sinus using purple anticoagulated blood collection tubes and subjected to centrifugation at 4°C and 3,500 rpm for 10 min to isolate plasma, which was then utilized for enzyme-linked immunosorbent assay (ELISA). Subsequently, mice were euthanized via cervical dislocation. Sections of the duodenum and ileum (1 ~ 2 cm) were collected for observation of small intestine morphology. Sections of the ileum and colon (1 ~ 2 cm), and cecum contents were obtained for the analysis of quantitative polymerase chain reaction (Q-PCR) and 16S rRNA sequencing, respectively.

### 2.2 Small intestine morphology analysis

Tissues from the duodenum and ileum were preserved in paraformaldehyde (4%) and then embedded in paraffin. Subsequently, paraffin-embedded sections with a thickness of 5 μm were stained with hematoxylin and eosin. Tissue morphology was observed using a microscope (Nikon Eclipse Ci-E, Japan) at 100× magnification, and images were taken with a digital microscope camera (Nikon DS-L4, Tokyo, Japan). Five well-formed villi and crypts were randomly selected from each image, and crypt depth and villi height were assessed utilizing ImageJ software for measurement.

### 2.3 ELISA analysis

Plasma concentrations of TNF-*α*, IL-6 and IL-1β were determined through ELISA procedures in accordance with the protocols provided by the respective manufacturer’s instructions (Cusabio, Wuhan, China). Hundred microliter of standard or sample was added to each well and then incubated at 37°C for 2 h. After removing the liquid, 100 μL of Biotin-antibody (1×) was added to each well and incubated at 37°C for 1 h. After aspirating and washing each well 3 times, 100 μL of HRP-avidin (1×) was added to each well and incubated at 37°C for 1 h. After aspirating and washing for 5 times, 90 μL of TMB Substrate was added to each well. Subsequently, the plate was incubated at 37°C for 20 min, which was protected from light. Finally, 50 μL of stop solution was added to each well, and a microplate reader (Multiskan 60, Thermo Fisher Scientific, Waltham, MA, United States) was used to measure the absorbance at 450 nm within 5 min.

### 2.4 Q-PCR analysis

Ileum and colon tissues were homogenized in l mL of Trizol reagent (Ambion, Austin, TX, USA) to extract total RNA. cDNA synthesis was carried out using the PreScript III RT Promix kit (Yingzan Biotechnology Co., Ltd., Guangzhou, China). Primer sequences are provided in Supplementary Table 1. Quantitative analysis was conducted using a Bio-Rad CFX96TM Real-Time System (Hercules, CA, United States) with a reaction mixture of 20 μL comprising 7 μL Nuclease-Free Water, 2 μL cDNA template, 10 μL 2× Robust SYBR Green qPCR ProMix (Inzan Biotechnology, Guangzhou, China), and 0.5 μL forward and reverse primer (10 μM). The PCR reaction was commenced with an initial denaturation phase at 95°C for 10 min, succeeded by 40 iterative cycles consisting of denaturation at 95°C for 5 s, and subsequent annealing/extension at 60°C for 20 s. The quantification of relative mRNA expression levels was executed employing the $2^{-\Delta \Delta CT}$ method, with *β*-actin serving as the internal reference gene for normalization.

### 2.5 Analysis of 16S rRNA sequencing

Cecum contents collected from four mice within each experimental group were dispatched to Kidio Biotechnology Co., Ltd. (Guangzhou, China) for the sequencing of bacterial 16S rRNA genes. The DNA extraction, amplicon purification and quantification performed using the method we previously reported (22). Briefly, total DNA from the samples was extracted using HiPure Stool NDA Kits (Magen, Guangzhou, China). The concentration and quality of DNA were obtained using the NanoDrop 2000 micro-spectrophotometer (Thermo Fisher Scientific, Waltham, MA, United States) and 1.2% agarose gels, respectively. The V3–V4 region of the bacterial 16S rRNA was amplified using the specific primers (341F: 5′-CCTACGG GNGGCWGCAG-3′, 806R: 3′-GGACTACHVGGGTWTCTAAT-5′) containing a barcode. Phusion High Fidelity PCR Master Mix (New England Biolabs, Inc., Ipswich, MA, United States) was used to perform the PCR reactions. The amplicons were extracted and purified using 2% agarose gels and AxyPrep DNA Gel Extraction Kit (Axygen Biosciences, Union City, CA, United States), respectively. The quantification of amplicons was performed using Qubitn3.0 Fluorometer (Thermo Fisher Scientific, United States) and ABI StepOnePlus Real-Time PCR System (Life Technologies, Foster City, United States). The quantified amplicons were sequenced on the Illumina MiSeq platform. Beta diversity analysis, encompassing NMDS, PCoA, and ANOSIM tests, was executed utilizing the Vegan package (version 2.5.3) within the R programming environment. Visualization of the results was achieved through implementation of the ggplot2 package.

### 2.6 Statistical analysis

The measurement data were depicted as mean ± standard deviation (SD) and subjected to analysis through one-way analysis of variance (ANOVA) in conjunction with the *Tukey test* for *post hoc* comparisons, employing SPSS Statistics 26.0. A threshold of *p* < 0.05 was set to determine statistical significance.

## 3 Results

### 3.1 AS restored small intestine morphology

**Figure 2:**
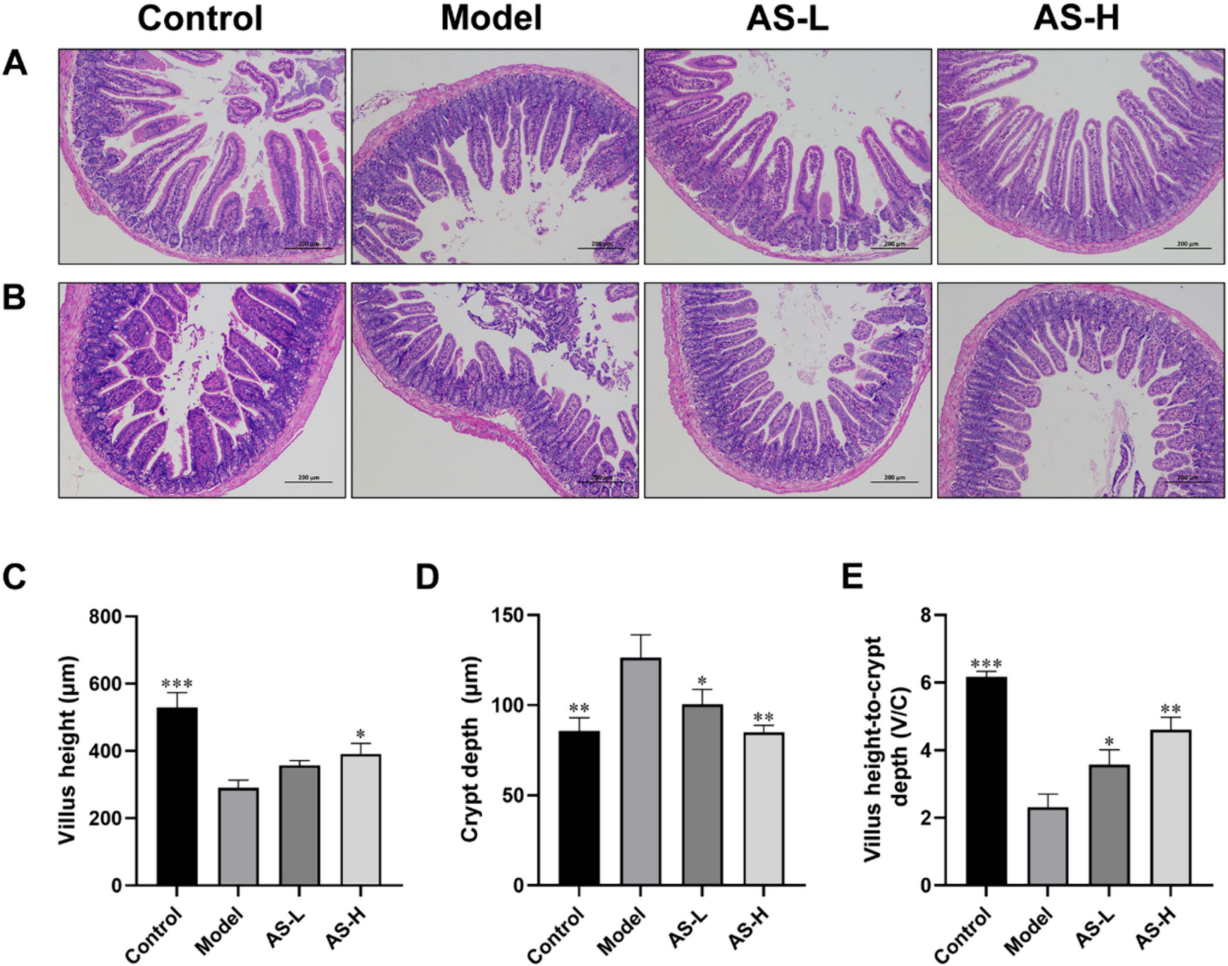
Astragalin (AS) improves small intestine morphology. Photographs of histopathological sections from the duodenum **(A)** and ileum **(B)** tissues by H&E staining of the four groups (magnification: 100×, scale bar: 200 μm). Villus height **(C)**. Crypt depth **(D)**. The ration of villus height to crypt depth **(E)**. The data are all expressed as mean ± SD, and the number of biological replicates was three (*n* = 3). *p < 0.05, **p < 0.01, ***p < 0.001 versus the Model group.

The structure of the small intestine was employed to assess the impairment of the IMB, with findings presented in Figure 2. Notably, LPS treatment induced a disruption in the integrity and arrangement of intestinal villi, resulting in a significant reduction in duodenal villi height (*p* < 0.001) and V/C value (*p* < 0.001), alongside an elevation in duodenal crypt depth (*p* < 0.01). However, AS treatment alleviated LPS-induced intestinal morphological disturbance, and increased villi height and V/C value (*p* < 0.05), while reducing crypt depth (*p* < 0.05). These findings suggest that AS treatment could restore small intestine morphology.

### 3.2 AS improved the expression of intestinal TJs and MUC2 mucin

**Figure 3:**
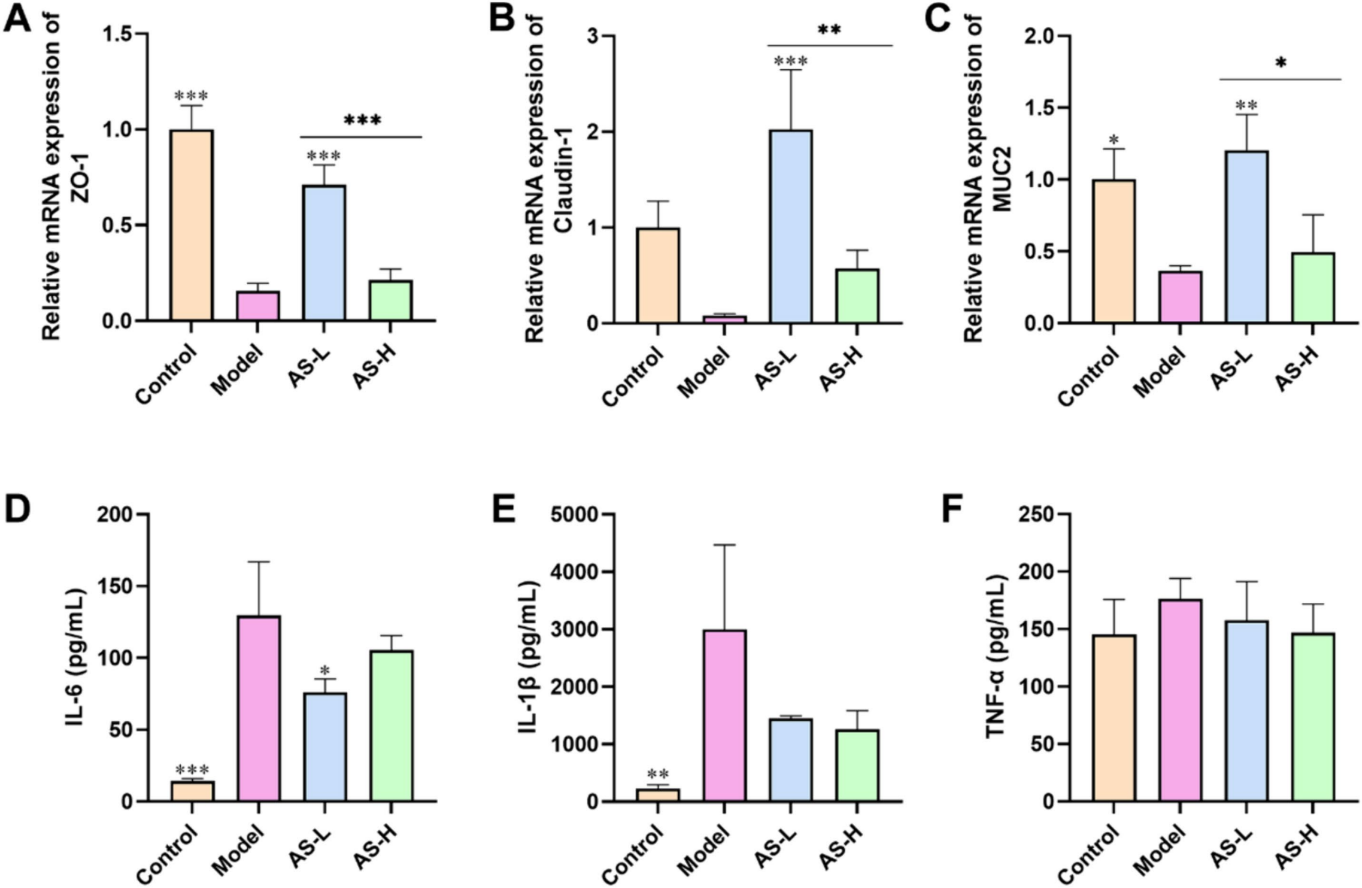
Astragalin improved the expression of intestinal TJs and MUC2 mucin, and reduced the levels of pro-inflammatory cytokines. The mRNA expression of ZO-1 **(A)**, Claudin-1 **(B)**, and MUC2 **(C)** in ileum tissues. The plasm levels of IL-6 **(D)**, IL-1β **(E)**, and TNF-*α* **(F)**. The data were all expressed as mean ± SD, and the number of biological replicates was three. *p < 0.05, **p < 0.01, ***p < 0.001 versus the Model group.

In this work, TJ proteins (ZO-1, Claudin-1) and MUC2 mucin were utilized to assess the impact of AS on the integrity of the IMB. Following LPS treatment, a decrease in the expression of ZO-1 (*p* < 0.001), Claudin-1, and MUC-2 (*p* < 0.05) was observed in the Model group compared to the Control group (Figures 3A–C). Conversely, mice treated with AS exhibited elevated expression levels of Claudin-1, ZO-1 and MUC-2 compared to the Model group, indicating improved integrity of the IMB. Notably, AS treatment at a dose of 50 mg/kg BW demonstrated higher expression levels of Claudin-1, ZO-1and MUC2 compared to that at 100 mg/kg BW (*p* < 0.05).

### 3.3 AS reduced the levels of pro-inflammatory cytokines

Furthermore, to evaluate the inflammatory status in mice, the plasma levels of pro-inflammatory cytokines were analyzed. As illustrated in Figures 3D–F, administration of LPS led to an increase in the plasma levels of IL-1β (*p* < 0.001), IL-6 (*p* < 0.01), and TNF-*α*. Conversely, treatment with AS resulted in a reduction levels of IL-6, IL-1β, and TNF-α relative to those observed in the Model group. These findings indicate that AS effectively mitigated the inflammatory responses triggered by LPS.

### 3.4 AS altered the composition of microbial communities

**Figure 4:**
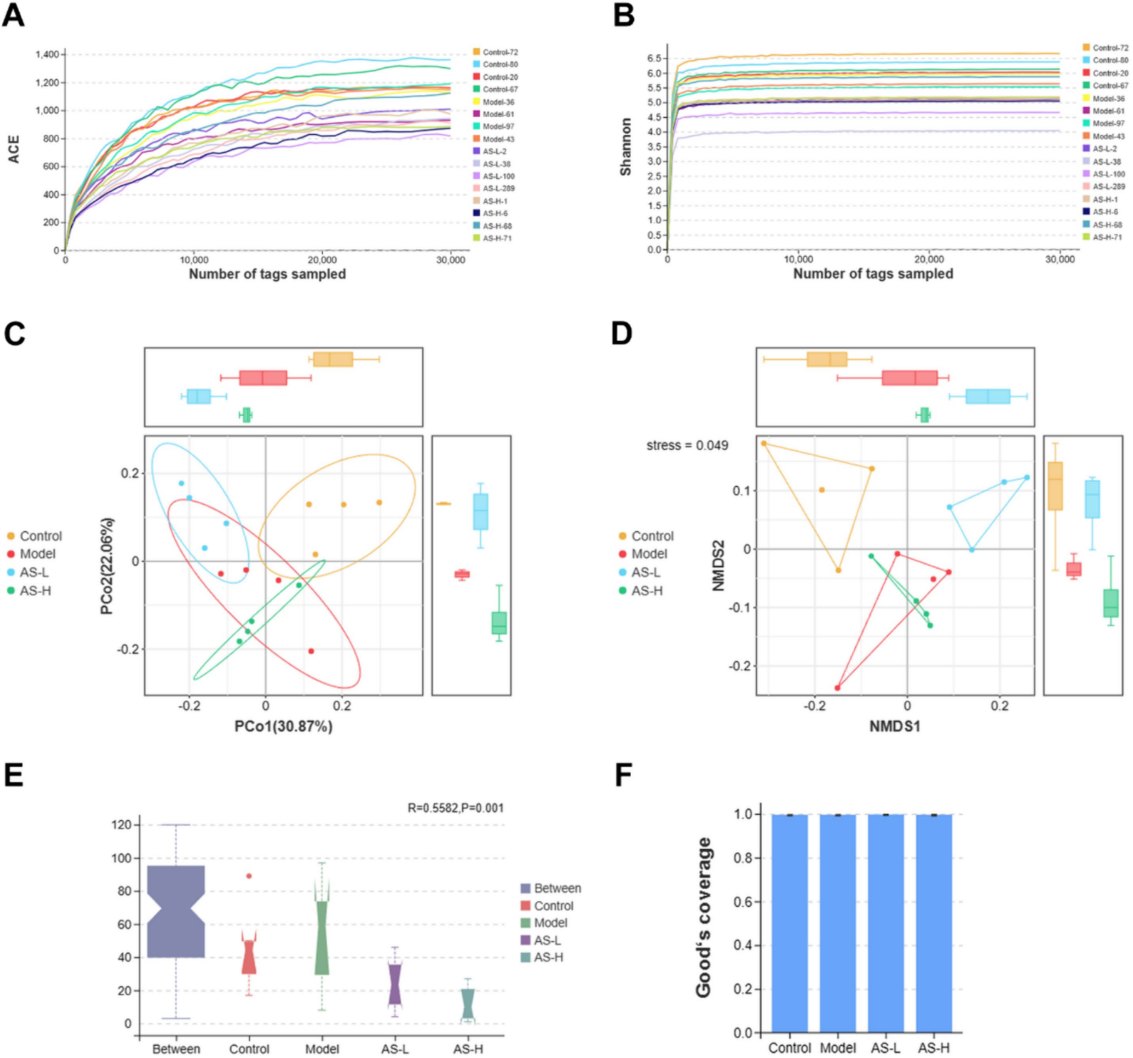
Astragalin modifies the diversity of the intestinal microbiota. The ACE **(A)** and Shannon **(B)** rarefaction curves. The PCoA **(C)** and NMDs **(D)** analysis based on Bray–Curtis distance. ANOSIM analysis **(E)**. Good’s coverage index **(F)**. Kruskal–Wallis *H* test was used to analyze the Bray–Curtis distance with a significant level of *p* < 0.05. And the *Tukey-HSD* test was used to analyze the Good’ s coverage index. The number of biological replicates was four (*n* = 4).

The impact of AS on the gut flora in mice was evaluated through 16S rRNA gene sequencing. Figure 4F illustrates that the sequencing coverage exceeded 99% for each group, ensuring the accuracy and reproducibility of the sequencing results. Figures 4A,B displays ACE and Shannon rarefaction curves reaching a plateau, indicating adequate and reasonable sampling of tags for sequencing. Additionally, Figure 4D indicates a stress value of 0.049 (<0.1) for the NMDs analysis, suggesting effective dimensionality reduction and accurate representation of the data distribution. Furthermore, the PCoA analysis in Figure 4C, based on Bray–Curtis distance, reveal distinct sample distributions among the four groups, indicating variations in intestinal flora between groups. ANOSIM analysis in Figure 4E demonstrates an *R*-value of 0.5582 (>0) and a *p*-value of 0.001 (<0.05), signifying a noteworthy disparity in microbial composition across the four groups. Thus, results from NMDs, PCoA, and ANOSIM analysis collectively indicate disparities in microbial composition among the experimental groups.

**Figure 5:**
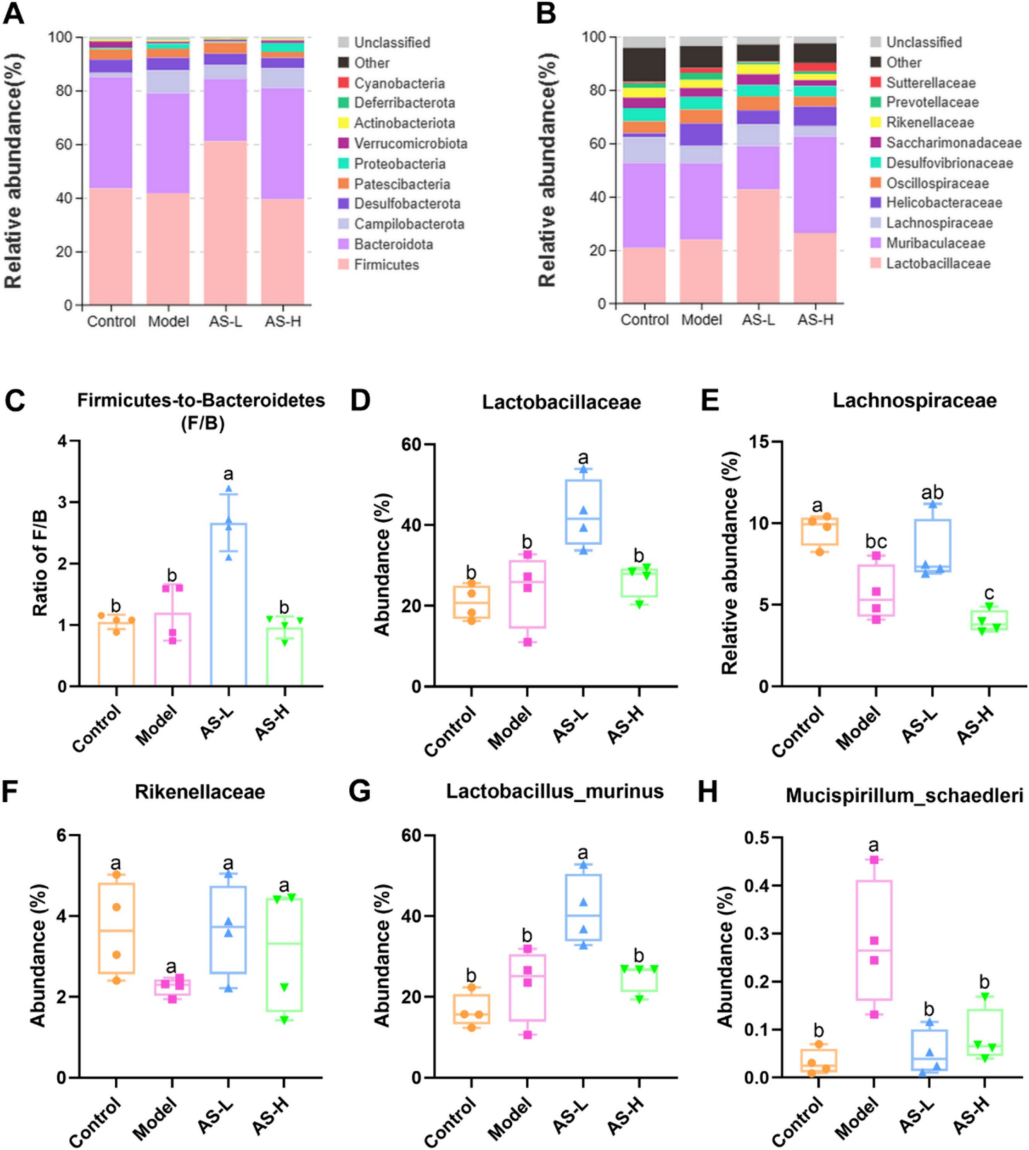
Astragalin alters the composition of intestinal microbiota. Intestinal microbiota composition at phylum **(A)** and family **(B)** levels. The ratio of Firmicutes to Bacteroidetes **(C)**. Changes of the major intestinal bacterial communities at family **(D–F)** and species **(G,H)** levels. The data of bacterial abundance were expressed as mean ± SD, and *Tukey HSD* test was used to analyze the statistical significance between groups. The number of biological replicates was four (*n* = 4). Different letters on the bars mean statistical difference with *p* < 0.05.

The composition of intestinal microorganisms in cecum contents among different groups was analyzed. Figure 5A illustrates the relative abundance of intestinal microbial communities at the phylum level, revealing 10 major phyla in cecum contents microbiota: *Firmicutes*, *Bacteroidota*, *Campilobacterota*, *Desulfobacterota*, *Patescibacteria*, *Proteobacteria*, *Verrucomicrobiota*, *Actinobacteriota*, *Deferribacterota*, and *Cyanobacteria*. Importantly, *Firmicutes* and *Bacteroidetes* emerged as the most abundant phyla across all four groups. Interestingly, Figure 5C highlights that AS treatment at a dose of 50 mg/kg BW significantly elevated the *Firmicutes* to *Bacteroidetes* ratio (F/B ratio). Moreover, as shown in Figure 5B, 10 major families were identified in the gut microbiota of mice, namely *Lactobacillaceae*, *Muribaculaceae*, *Lachnospiraceae*, *Helicobacteraceae*, *Oscillospiraceae*, *Desulfovibrionaceae*, *Saccharimonadaceae*, *Rikenellaceae*, *Prevotellaceae*, and *Sutterellaceae*. Compared with the Model group, AS treatment (at 50 mg/kg BW) increased the relative abundances of *Lactobacillaceae* (*p* < 0.05), *Lachnospiraceae*, and *Rikenellace* (Figures 5D–F). Further analysis of the intestinal microbial community richness was performed at the species level (Figures 5G,H). It can be found that AS treatment (at 50 mg/kg BW) significantly increased the relative abundance of *Lactobacillus murinus* (*L. murinus*) (*p* < 0.05). Furthermore, the relative abundance of *Mucispirillum schaedleri* (*M. schaedleri*) was raised in the Model group (*p* < 0.05), while AS treatment reduced the relative abundance of *M. schaedleri* (*p* < 0.05).

### 3.5 AS altered biomarkers in the microbial communities

**Figure 6:**
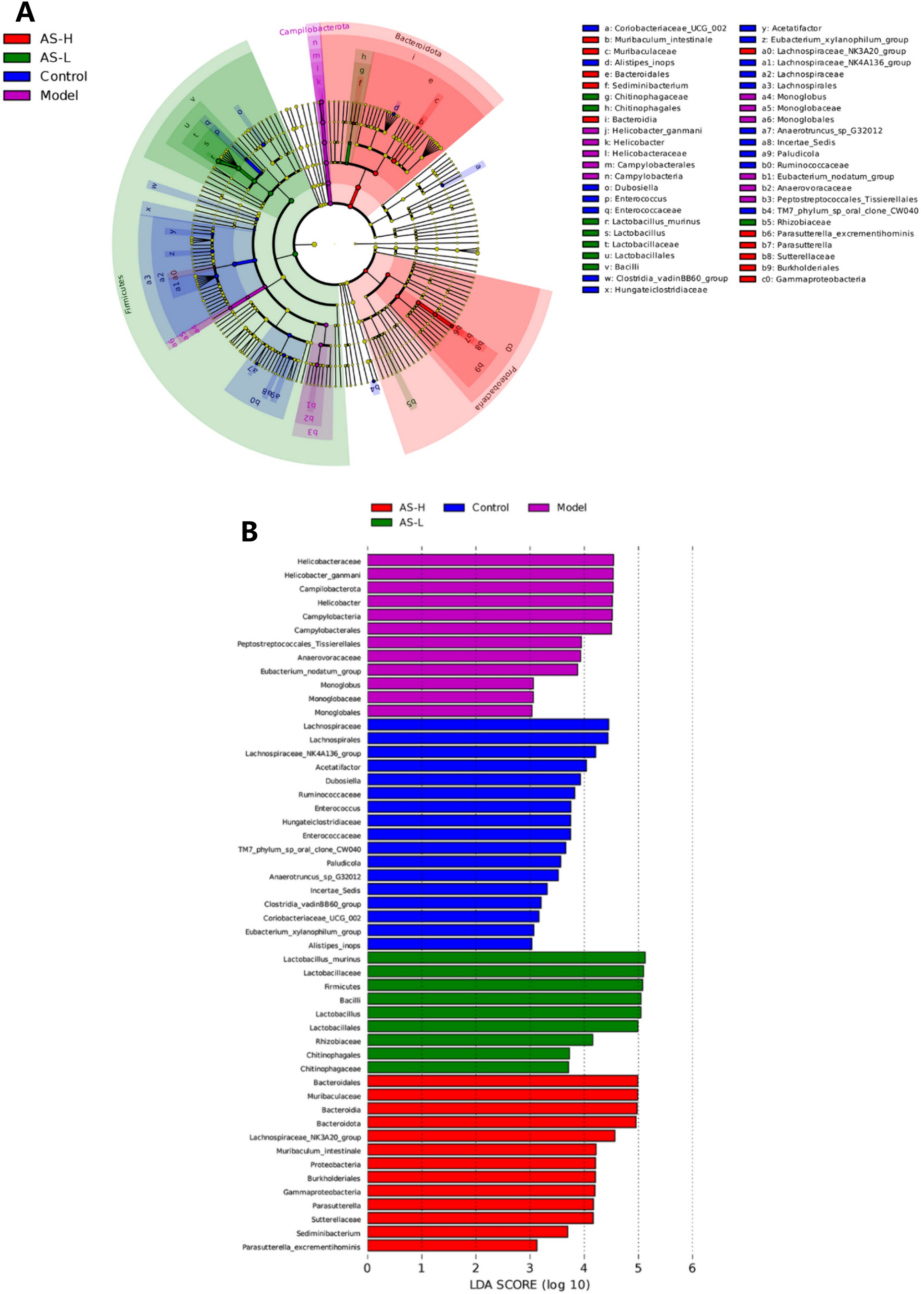
Cladogram of OTUs (LDA > 3) **(A)**, and the six circles represents the phylum, class, order, family, genus, and species classification levels from the inside to the outside, respectively. Barplot of LEfSe analysis (LDA > 3) **(B)**, the Wilcoxon and Kruskal–Wallis tests were used to screen for dominant microbial communities of the four groups with significant differences at a level of *p* < 0.05.

Linear discriminant analysis effect size (LEfSe) analysis was used to further assess the effect of LPS and AS treatment on the composition of the intestinal microbiota of mice. A cladogram was constructed to pinpoint biomarkers that demonstrated significant variations in abundance across the different groups. As depicted in Figure 6A, the five circles, ranging from the inside to the outside, represent the classifications at the phylum, class, order, family, genus, and species levels. Each small circle within a concentric layer signifies a taxonomic classification at that specific level, where the diameter of the circles is directly proportional to the relative abundance of the classification (23). The small yellow circles indicate no significant differences in abundance between groups for that classification. It is evident that there exists a distinct variation in the representative microbiota present within each group of mice. Notably, there were 7, 15, 11, 10, 4, and 4 different abundance taxonomic clades with an LDA > 3 at the species, genus, family, order, class, and phylum levels, respectively (Figure 6B). Specifically, *L. murinus*, *Lactobacillus*, and *Lactobacillaceae* from *Lactobacillales*, *Bacilli* from *Firmicutes*, and *Rhizobiaceae* from *Proteobacteria* were enriched in the group receiving AS treatment at a dosage of 50 mg/kg BW (LDA > 4). *Helicobacter ganmani*, *Helicobacter*, *Helicobacteraceae*, *Campylobacterales* and *Campylobacteria* from *Campilobacterota* were enriched in the Model group (LDA > 4). *Lachnospirales*, *Lachnospirales*, *Lachnospiraceae NK4A136 group* and *Acetatifactor* from *Firmicutes* were enriched in the Control group (LDA > 4). *Bacteroidia*, *Bacteroidia*, *Muribaculum intestinale* from *Bacteroidota*, *Lachnospiraceae NK3A20 group* from *Firmicute*, and *Burkholderiales*, *Gammaproteobacteria*, *Parasutterella*, *Sutterellaceae* from *Proteobacteria* were enriched in the group of AS treatment at a dose of 100 mg/kg BW. In summary, both LPS and AS treatment induced changes in gut microbiota biomarkers in mice.

## 4 Discussion

In the intricate balance that underpins gut health and overall wellbeing, the integrity and functionality of the IMB emerges as a cornerstone. This fine-tuned membrane, gracefully lining the small intestine, not only acts as a vigilant gatekeeper, selectively allowing nutrients to pass through, but also actively facilitates their efficient absorption (24). Disruptions to this intricate equilibrium have far-reaching consequences, disrupting gut homeostasis, paving the way for pathogenic microorganisms to infiltrate, and triggering inflammatory cascades that can spiral into various intestinal ailments. Given this context, it is of paramount importance to unravel the modulatory influence that external stimuli, like LPS, and potential therapeutic interventions, such as AS, exert on intestinal morphology. Our study thus embarked on a comprehensive evaluation of the multifaceted effects of AS in mice with LPS-induced IMB injury, exploring four vital aspects: firstly, the structural alterations within the intestinal mucosa; secondly, the regulation of TJs and MUC2 expression, key players in maintaining barrier integrity; thirdly, the systemic modulation of pro-inflammatory cytokines in the plasma, indicative of immune responses; and finally, the shifts in the composition of the intestinal microbiota, a pivotal aspect of gut health. Through this holistic approach, we aimed to gain a deeper insight into how AS may restore and enhance the functionality of the IMB, mitigating the damaging effects of LPS.

The morphology of the intestinal mucosa plays a crucial role in nutrient absorption within the small intestine (24). Disruption of intestinal morphology, such as villi loss, villi atrophy, and crypt hyperplasia, may contribute to the invasion of pathogenic bacteria and subsequently induce various intestinal inflammatory diseases (25). Damage to the IMB induced by LPS often coincides with alterations in intestinal morphology, such as decreased villi height, increased crypt depth, and a reduced ratio of villi height to crypt depth (26). In our study, LPS administration resulted in decreased villus height and V/C ratio, accompanied by an elevation in crypt depth, consistent with findings reported by Yang et al. (27) and Yu et al. (28), indicating the induction of damage in the IMB. An increase in villi height can enhance surface area for nutrient absorption, thereby improving nutrient digestibility (29). Furthermore, the elevated ratio of villus height to crypt depth suggests a heightened turnover of intestinal epithelial cells and enhanced capacity for nutrient absorption (30). Mice treated with 50 and 100 mg/kg AS showed increased villus height and V/C, as well as reduced crypt depth, which indicated that AS restored intestinal morphological disorder induced by LPS, thereby enhancing intestinal mucosal function and alleviating intestinal mucosal damage.

Tight junctions, composed primarily of Claudins, Occludin, and scaffolding proteins such as Zonula Occludens (ZO) proteins 1–3, are crucial for maintaining the function and integrity of the IMB (30, 31). Goblet cells in IECs secrete MUC2, the most important component of the intestinal mucus layer, which shields the intestine from harmful microorganisms and food antigens (32). Moreover, LPS serves as a potent stimulus for the immune system, eliciting a robust innate immune response in the organism while potentially causing damage to the intestinal mucosa (33). Studies by Xiong et al. (34) have revealed that LPS stimulation induces IMB injury, resulting in decreased mRNA expression levels of Claudin-1, Occludin, and ZO-1, coupled with a concomitant increase in pro-inflammatory cytokines, including IL-1β, TNF-*α*, and IL-6. In line with previous findings, our study utilizing C57BL/6J mice demonstrated that LPS treatment resulted in a marked decrease in the expression levels of ZO-1 and Claudin-1, as well as MUC2 mucin in ileal tissue. This was accompanied by an increase in plasma levels of IL-6, IL-1β, and TNF-*α*, thereby indicating the successful establishment of a mouse model with induced damage to the IMB. Of importance, ZO-1 plays a crucial role in IMB repair, being the first identified TJ protein that binds to various other proteins including Claudins and Occludin (35). Additionally, Claudin-1 is vital for maintaining the structure and physiological function of the intestinal mucosa (36). In our study, mice treated with AS exhibited upregulated expression of Claudin-1, ZO-1, and MUC2, alongside downregulated levels of IL-6, IL-1β, and TNF-*α*, suggesting improvement in IMB function and reduction in inflammatory responses. These findings are consistent with the results of Peng et al., who demonstrated that AS improves IMB function in DSS-induced colitis by increasing the expression of Occludin, ZO-1, and MUC2 (37). AS, being a natural flavonoid with anti-inflammatory activity, can attenuate the LPS-induced inflammatory response in mice by downregulating the production of IL-6, IL-1, and TNF-α through inhibition of the NF-κB signaling pathway (38), aligning with the observations in our study. Interestingly, AS treatment at a dose of 50 mg/kg exhibited higher expression levels of Claudin-1, ZO-1, and MUC2 compared to 100 mg/kg, indicating better amelioration of LPS-induced disruption of intestinal mucosal integrity. These results collectively suggest that AS enhances IMB function by alleviating inflammatory responses.

The intestinal microbiota is of great importance in human health and the development of disease (39). The dysfunctional composition and function of the intestinal microbiota can disrupt intestinal homeostasis, which can be detrimental to intestinal health (40). Flavonoids can effectively ameliorate several intestinal diseases by regulating intestinal flora (41). However, despite being a flavonoid widely present in edible plants, few studies have been reported on the effects of AS on the regulation of intestinal flora. Therefore, to delve deeper into the potential involvement of intestinal flora in the mechanism underlying AS-mediated alleviation of LPS-induced IMB damage, the 16S rRNA gene sequencing method was employed to scrutinize alterations in intestinal flora composition in mice throughout the process.

The normal intestinal microbiota in humans and mice is composed of two major phyla, namely *Bacteroidetes* and *Firmicutes* (42). In this work, it was also found that the relative abundance of *Firmicutes* and *Bacteroidetes* ranked in the top two in all four groups at the phylum level. Particularly, AS treatment at a dose of 50 mg/kg significantly increased the F/B ratio. Consistent with the results of this study, Qu et al. found that kaempferol, a natural flavonoid found in numerous medicinal plants, could significantly increase the F/B ratio to remodel the intestinal flora of mice with ulcerative colitis, which helped to ameliorate intestinal damage (43). Additionally, AS treatment at a dose of 50 mg/kg BW increased the relative abundance of *Lachnospiraceae* compared to the Model group. *Lachnospiraceae*, which can produce butyrate, is commonly found in the intestines of humans (44). Butyrate serves as a vital energy source for intestinal epithelial cells, facilitating mucin secretion, mitigating intestinal inflammation, and bolstering intestinal immunity, thereby safeguarding the IMB (45). Furthermore, as per the outcomes of LEfSe analysis (depicted in Figure 6), the advantageous bacteria *Lachnospiraceae NK3A20* group exhibited enrichment in mice treated with AS at a dosage of 100 mg/kg BW (LDA > 4). Prior research has elucidated that the *Lachnospiraceae NK3A20* group plays a role in the degradation of indigestible plant cellulose and hemicellulose within the host intestinal tract, yielding short-chain fatty acids that contribute to intestinal health maintenance (46).

Moreover, mice treated with AS at a dose of 50 mg/kg BW showed a higher abundance of *L. murinus* as compared with that in the other three groups. Notably, *Lactobacillus* is an intestinal probiotic that can regulate intestinal epithelial function and increase the formation of TJs (47). Thus, *Lactobacillus* plays a key role in the intestine as the commensal bacteria. *L. murinus* from *Lactobacillus* is reportedly a potential probiotic that plays an important part in alleviating intestinal inflammation by regulating intestinal immunity (48). Pan et al. (49) found that *L. murinus* was a commonly identified strain of *Lactobacillus* with the most pronounced anti-colitis effect, which could improve IMB damage effectively. Thus, AS potentially mitigates IMB damage by fostering the proliferation of *L. murinus* from *Lactobacillus*. In addition, AS treatment reduced the relative abundance of *M. schaedleri*. Importantly, *M. schaedleri* typically inhabits the mucus layer of the intestinal mucosa in minimal quantities and possesses a substantial pathogenic potential for instigating intestinal inflammation (50). These findings suggest that AS enhances the intestinal micro-ecological environment chiefly by fostering the growth of beneficial intestinal microbiota such as *Lachnospiracea* and *L. murinus*, while curtailing the relative abundance of pathogenic bacteria like *M. schaedleri*, thereby preserving the integrity of the IMB. Remarkably, the results also indicate that a dosage of 50 mg/kg BW of AS treatment exhibits superior remodeling effects on gut flora compared to 100 mg/kg BW. Further investigations are warranted to elucidate the mechanisms through which AS enhances the IMB in mice via modulation of the gut microbiota.

## 5 Conclusion

**Figure 7:**
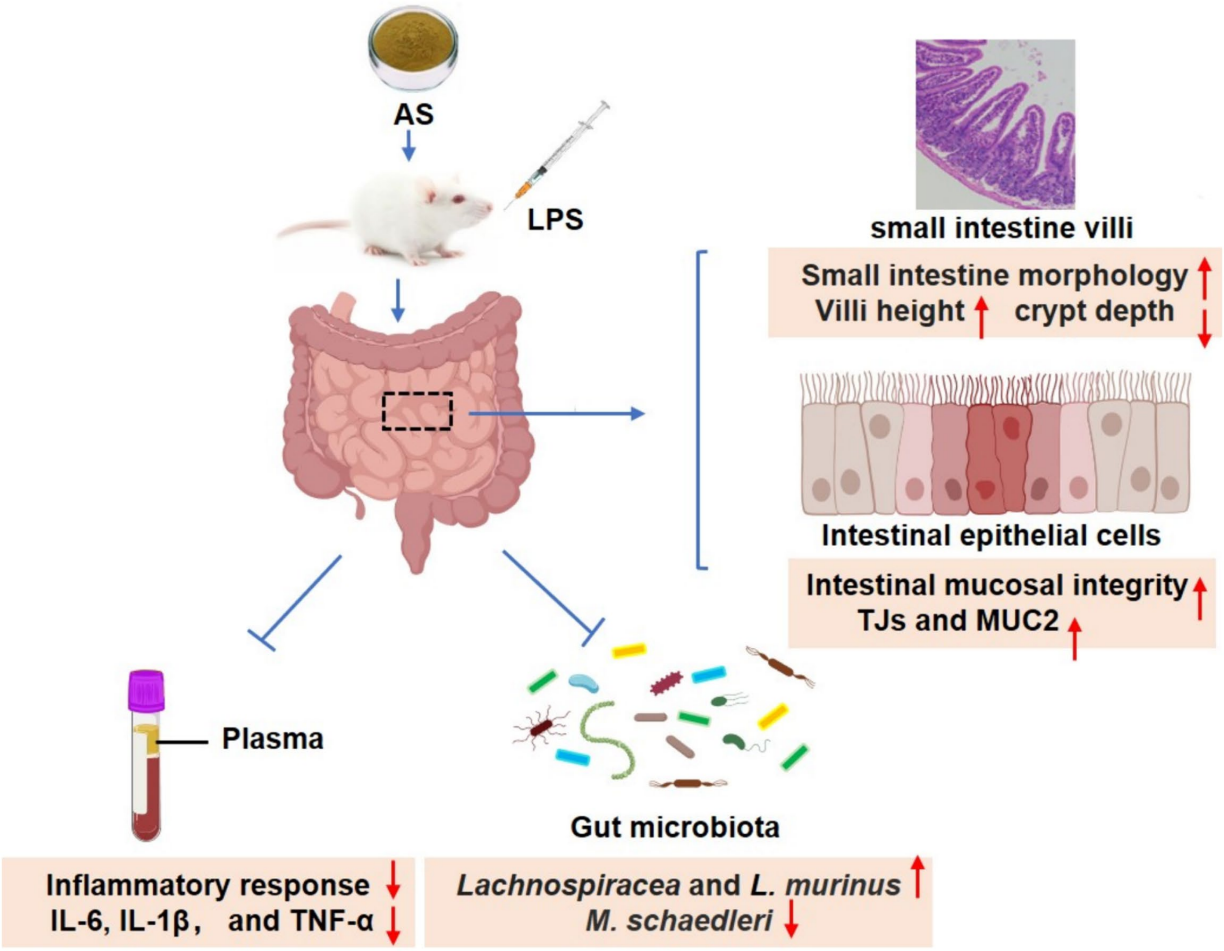
Astragalin ameliorates LPS-induced intestinal mucosal barrier damage by mitigating serum inflammatory factors and modulating gut microbiota in mice.

The results of this investigation propose that AS can protect against the IMB damage, as indicated by improvements in small intestine morphology and enhanced the IMB function. Notably, AS appears to exert its protective mechanisms by reducing inflammation and influencing the composition of the intestinal microbiota (Figure 7). Interestingly, the dose of 50 mg/kg BW of AS exhibits better modulation of intestinal flora composition and more effectively ameliorates LPS-induced IMB damage compared to 100 mg/kg BW. These study findings offer a theoretical foundation for the management of IMB damage and the development of AS-related nutraceuticals.

## Data Availability

The original contributions presented in the study are included in the article/Supplementary material, further inquiries can be directed to the corresponding authors.
