# Supplementary material for: Dietary astragalin confers protection against lipopolysaccharide-induced intestinal mucosal barrier damage through mitigating inflammation and modulating intestinal microbiota
Source: Front Nutr. 2024 Oct 2;11:1481203. doi: 10.3389/fnut.2024.1481203 (PMC11483603; doi:10.3389/fnut.2024.1481203)
Supplement: Supplementary file 1 [file Table_1.pdf]

Table 1 Primer sequences for Q-PCR analysis

**Target gene**	**Forward sequence (5′ → 3′)**	**Reverse sequence (3′ → 5′)**
ZO-1	GCTTTAGCGAACAGAAGGAGC	TTCATTTTTCCGAGAAGGAGC
Claudin-1	TCAGGTCTGGCGACATTAGT	GACAGGAGCAGGAAAGTAGGA
Mucin-2	ATGCCCACCTCCTCAAAGAC	GTAGTTTCCGTTGGAACAGTGAA
