# Supplementary material for: Dietary astragalin confers protection against lipopolysaccharide-induced intestinal mucosal barrier damage through mitigating inflammation and modulating intestinal microbiota
Source: Front Nutr. 2024 Oct 2;11:1481203. doi: 10.3389/fnut.2024.1481203 (PMC11483603; doi:10.3389/fnut.2024.1481203)
Supplement: Supplementary file 2 [file Data_Sheet_1.pdf]

Raw data

1. small intestine morphology

Control-duodenum：

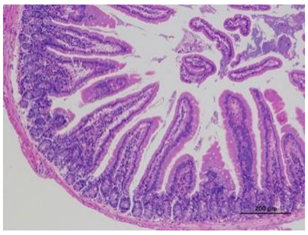

Control-ileum：

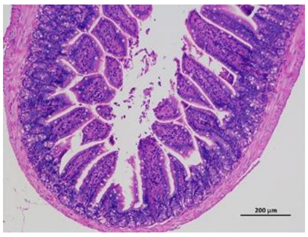

Model-duodenum：

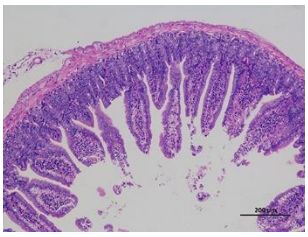

Model-ileum：

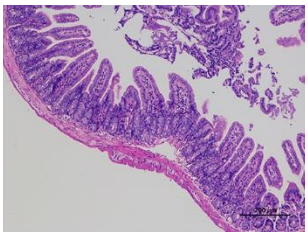

AS-L-duodenum：

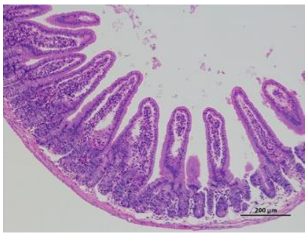

AS-L-ileum：

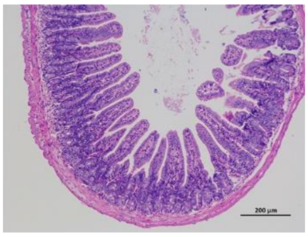

AS -H-duodenum:

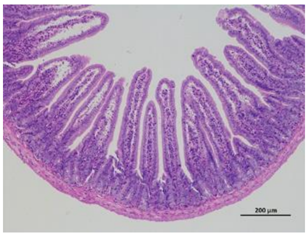

AS -H-ileum:

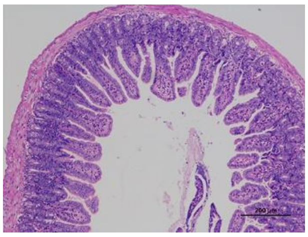

2. Villus height and crypt depth

GROUP	NO.	Villus height (μm)	Average of villus height (μm)	Crypt depth (μm)	Average of crypt depth (μm)	Villus height/Crypt depth
Control	72	506.028	507.14	96.167	84.44	6.01
		513.256		81.707		
		477.297		83.546		
		464.096		82.389		
		575.044		78.409		
	20	529.120	500.15	76.551	79.02	6.33
		533.014		70.880		
		453.542		84.593		
		483.01		73.756		
		502.06		89.308		
	80	600.004	580.08	98.489	93.66	6.19
		582.718		89.889		
		513.069		113.772		
		615.38		89.554		
		589.227		76.577		
Model	75	325.576	314.98	144.9	117.41	2.68
		312.87		104		
		278.568		97.015		
		314		114.56		
		343.89		126.57		
	97	236.008	285.11	131.605	120.80	2.36
		272.617		118.068		
		290.682		115.879		
		286.252		110.055		
		340		128.39		
	43	308.759	270.98	135.204	140.87	1.92
		262.084		141.718		
		268.425		133.507		
		256.32		144		
		259.307		149.933		
AS-L	38	323.048	357.54	112.089	104.60	3.42
		397.683		89.844		
		374.342		115.948		
		360.494		101.272		
		332.151		103.846		
	2	347.039	342.48	90.355	106.04	3.23
		326.166		110.923		
		374.604		113.701		

		340.588		93.744		
		324.025		121.458		
	74	371.137	370.76	81.621	91.15	4.07
		400.597		104.371		
		371.006		78.991		
		346.700		95.356		
		364.348		95.392		
AS-H	1	325.208	372.93	92.477	88.16	4.23
		334.527		93.059		
		408		80.994		
		407.922		97.324		
		388.973		76.942		
	42	471.071	427.41	88.814	85.98	4.97
		410.59		84.380		
		422.298		86.093		
		389.225		90.973		
		443.842		79.649		
	63	376.34	372.24	82.000	80.68	4.61
		407.671		62.032		
		362.480		93.059		
		407.671		74.243		
		307.044		92.087		

3. Relative mRNA expression levels of intestinal tight junctions

ZO-1：

	β-actin CT			CT-Mean	ZO-1 CT			CT Mean
Control	22.32	22.23	22.35	22.30	27.99	27.9	27.77	27.89
	22.76	22.73	22.66	22.72	28.52	28.62	28.6	28.58
	23.14	23.51	23.2	23.28	28.72	28.69	28.93	28.78
Model	21.66	21.84	22.05	21.85	29.82	29.77	29.95	29.85
	22.49	22.55	22.55	22.53	31.03	30.91	30.73	30.89
	22.42	22.47	22.62	22.50	31.2	31.19	31.15	31.18
AS-L	21.42	21.25	21.25	21.31	27.98	27.35	27.79	27.71
	21.18	21.10	21.07	21.12	27.22	27.02	27.08	27.11
	21.13	21.15	21.11	21.13	27.2	27.16	27.12	27.16
AS-H	21.09	21.04	21.09	21.07	28.54	28.55	28.87	28.65
	21.39	21.4	21.34	21.38	29.12	29.17	29.13	29.14
	20.64	20.55	20.65	20.61	29.02	28.89	29.08	29.00

MUC2：

	βCT			CT Mean	Mucin-2 CT			CT Mean
Control	22.36	22.28	22.19	22.28	26.53	26.71	26.61	26.62
	22.32	22.23	22.35	22.30	27.11	27.28	27.24	27.21
	21.91	22.03	22.01	21.98	26.36	26.32	26.33	26.34
Model	22.03	21.75	21.89	21.89	27.8	27.93	27.94	27.89
	22.49	22.55	22.55	22.53	28.7	28.63	28.65	28.66
	21.92	21.97	22.12	22.00	27.87	27.83	27.77	27.82
AS-L	21.18	21.10	21.07	21.12	25.09	25.06	25.01	25.05
	21.13	21.15	21.11	21.13	25.59	25.59	25.56	25.58
	21.73	21.63	21.76	21.71	26.04	26.1	26.2	26.11
AS-H	20.97	20.92	20.6	20.83	26.81	26.74	26.58	26.71
	21.29	21.49	21.53	21.44	27.99	27.38	27.71	27.69
	20.64	20.55	20.65	20.61	25.28	25.5	25.62	25.47

Claudin-1:

	βCT			CT Mean	C1 CT			CT Mean
Control								
	18.42	18.44	18.43	18.43	31.63	32.08	32.63	32.11
	18.35	19.15	18.87	18.79	31.39	31.97	31.83	31.73
	19.26	19.23	19.16	19.22	31.89	32.33	32.14	32.12
Model	17.86	17.88	17.95	17.90	34.31	34.98	35.01	34.77
	18.02	18.08	18.05	18.05	34.95	34.98	34.84	34.92
	18.08	17.93	18.1	18.04	34.94	34.27	34.13	34.45
AS-L	18.59	18.97	18.88	18.81	30.77	30.58	30.76	30.70
	19.19	19.45	19.81	19.48	31.49	31.35	31.23	31.36
	18.73	18.63	18.76	18.71	31.43	31.7	31.22	31.45
AS-H	19.78	19.71	19.77	19.75	33.39	33.46	33.16	33.34
	19.54	19.53	19.61	19.56	34.11	33.99	34.4	34.17
	20.29	20.49	20.53	20.44	34.3	34.28	34.14	34.24

4. Plasma levels of IL-6, IL-1β, and TNF-α：

TNF-α			
GROUP	NO.	concentration（pg/ml）	Abs
standard solution	S0	0	0.071
	S1	15.6	0.123
	S2	31.2	0.21
	S3	62.5	0.387
	S4	125	0.805
	S5	250	1.252
Control	16	178.41	0.886
	49	138.82	0.692
	72	118.41	0.592
Model	75	178.61	0.887
	50	192.90	0.957
	87	157.59	0.784
AS-L	64	120.04	0.600
	44	167.39	0.832
	63	185.35	0.920
AS-H	6	168.20	0.836
	63	119.22	0.596
	37	152.69	0.760

IL-6			
GROUP	NO.	concentration（pg/ml）	Abs
standard solution	S0	0.078	0
	S1	0.106	1.56
	S2	0.129	3.12
	S3	0.18	6.25
	S4	0.287	12.5
	S5	0.529	25
Control	72	0.218	12.44
	20	0.258	14.65
	80	0.276	15.65
Model	75	1.638	90.90
	50	2.984	165.26
	87	2.396	132.77
AS-L	100	1.298	72.11
	2	1.564	86.81
	83	1.250	69.46
AS-H	1	1.724	95.65
	81	2.092	115.98
	46	1.890	104.82

IL-1β			
GROUP	NO.	concentration（pg/ml）	Abs
standard solution	S0	0	0.071
	S1	31.25	0.123
	S2	62.5	0.21
	S3	125	0.387
	S4	250	0.805
	S5	500	1.252
Control	67	209.27	0.26
	8	172.00	0.219
	49	300.18	0.360
Model	87	3,583.82	3.972
	61	4,085.64	4.524
	43	1,329.27	1.492
AS-L	289	1,409.27	1.580
	38	1,467.45	1.644
	74	1,485.64	1.664
AS-H	71	1,403.82	1.574
	68	889.27	1.008
	6	1,489.27	1.668

5. Intestinal microbiota

5.1 Intestinal microbiota composition at family level

Family	Control-72	Control-80	Control-20	Control-67	Model-36	Model-61	Model-97	Model-43	AS-L-2	AS-L-38	AS-L-100	AS-L-289	AS-H-1	AS-H-6	AS-H-68	AS-H-71
Lactobacillaceae	16.2954	18.3482	23.0953	25.6297	11.0003	32.7179	27.2741	24.4461	39.3974	53.9171	43.7249	33.7039	27.4117	29.4963	20.2571	28.4257
Muribaculaceae	29.6702	29.9386	34.3071	33.2742	37.5201	20.7784	38.06	18.4911	21.195	15.6821	12.748	15.3368	32.9485	38.2265	39.3475	33.8869
Lachnospiraceae	10.4165	9.7823	10.1025	8.2371	8.0217	4.7974	4.0876	8.6293	7.4649	6.9173	11.1816	7.2223	3.368	4.8919	3.5972	3.9933
Helicobacteraceae	2.5432	1.2705	1.3346	0.7755	3.8362	13.0301	2.9804	14.0485	6.0296	2.2179	2.2685	10.2558	8.9604	6.4622	6.0495	7.7098
Oscillospiraceae	6.6552	3.2124	5.5175	2.5323	6.84	4.2022	3.58	6.0476	6.462	2.6091	3.8806	7.9887	3.1744	3.3235	5.0986	3.3899
Desulfovibrionaceae	2.6664	7.9129	5.2722	4.0166	3.3783	3.7537	5.9341	5.772	2.9437	4.3203	6.8595	2.6608	4.5349	2.7908	5.0821	3.1816
Saccharimonadaceae	4.3235	4.442	2.7333	4.0662	4.952	3.8229	1.8714	2.961	2.8605	4.8287	2.8267	5.8992	2.0738	1.8426	2.2256	2.9234
Rikenellaceae	4.2218	5.0279	3.0449	2.4027	1.9424	2.2755	2.469	5.2781	3.5873	2.2179	5.0478	3.8725	1.416	2.2323	4.4073	1.1632
Prevotellaceae	3.8711	1.1012	0.7049	1.1375	3.8673	3.0146	2.1625	1.7351	0.203	1.2322	1.1046	0.2759	0.4176	0.4532	1.9751	0.706
Sutterellaceae	0.3507	0.4464	0.4928	0.825	3.3453	1.3343	1.6664	0.5622	0.2619	0.3791	0.181	0.4518	5.6336	1.0595	1.0643	4.9761
Other	14.6358	13.8597	9.5876	13.0156	11.8909	7.5379	7.0697	6.5985	6.9698	3.8598	7.9593	7.0397	7.6966	7.5222	7.3551	7.0444
Unclassified	4.3502	4.6577	3.8073	4.0871	3.4054	2.735	2.8444	5.4302	2.625	1.8181	2.2177	5.2925	2.3642	1.6996	3.5405	2.6

Domain	Phylum	Class	Order
Bacteria	Firmicutes	Bacilli	Lactobacillales
Bacteria	Bacteroidota	Bacteroidia	Bacteroidales
Bacteria	Firmicutes	Clostridia	Lachnospirales
Bacteria	Campilobacterota	Campylobacteria	Campylobacterales
Bacteria	Firmicutes	Clostridia	Oscillospirales
Bacteria	Desulfobacterota	Desulfovibrionia	Desulfovibrionales
Bacteria	Patescibacteria	Saccharimonadia	Saccharimonadales
Bacteria	Bacteroidota	Bacteroidia	Bacteroidales
Bacteria	Bacteroidota	Bacteroidia	Bacteroidales
Bacteria	Proteobacteria	Gammaproteobacteria	Burkholderiales

5.2 Intestinal microbiota composition at Phylum level

Phylum	Control-72	Control-80	Control-20	Control-67	Model-36	Model-61	Model-97	Model-43
Firmicutes	40.7051	42.6264	45.0084	45.3698	34.4891	46.8303	39.4142	45.8165
Bacteroidota	46.1637	39.401	41.6453	39.202	46.4927	29.2243	44.8407	28.8004
Campilobacterota	2.5432	1.2705	1.3346	0.7755	3.8362	13.0301	2.9804	14.0485
Desulfobacterota	2.6691	7.9148	5.2722	4.0166	3.3783	3.7537	5.9341	5.772
Patescibacteria	4.3235	4.442	2.7378	4.0662	4.952	3.8229	1.8714	2.961
Proteobacteria	0.3882	0.4911	0.6165	0.8708	3.551	1.3952	1.7488	0.6416
Verrucomicrobiota	1.9302	1.9345	1.6749	3.687	1.4495	0.4069	0.6627	0.5093

Actinobacteriota	0.2088	0.6957	0.6806	0.8803	0.3473	0.3239	1.2853	0.2249
Deferribacterota	0.0696	0.0186	0.0088	0.0305	0.2445	0.454	0.2854	0.0529
Cyanobacteria	0.0134	0.0074	0.0044	0.0514	0.0602	0.0221	0.0096	0.022
Other	0.0054	0.013	0.011	0.0133	0	0.0138	0.0191	0.0198
Unclassified	0.9798	1.1849	1.0054	1.0365	1.1992	0.7225	0.9481	1.131
Phylum	AS-L-2	AS-L-38	AS-L-100	AS-L-289	AS-H-1	AS-H-6	AS-H-68	AS-H-71
Firmicutes	57.809	66.3145	63.2682	56.7794	39.3529	41.9059	34.5873	41.4132
Bacteroidota	27.456	20.5263	23.2479	21.8346	36.9062	42.3869	49.274	37.9331
Campilobacterota	6.0296	2.2179	2.2685	10.2558	8.9604	6.4622	6.0495	7.7098
Desulfobacterota	2.9437	4.3203	6.8595	2.6608	4.5349	2.7908	5.0821	3.1816
Patescibacteria	2.8605	4.8287	2.8267	5.8992	2.0738	1.8426	2.2256	2.9234
Proteobacteria	0.3228	0.4257	0.3011	0.5615	5.6928	1.135	1.1265	5.0694
Verrucomicrobiota	1.1572	0.3481	0.2182	1.1924	0.9643	2.0951	0.3365	0.5443
Actinobacteriota	0.7187	0.4033	0.4601	0.1985	0.3782	0.9005	0.4371	0.2115
Deferribacterota	0.0102	0.0241	0.181	0.1162	0.1685	0.0398	0.0622	0.0684
Cyanobacteria	0.0102	0.0086	0.0271	0.0242	0.0036	0.0139	0.0293	0.0093
Other	0.0061	0.0069	0.0085	0.0145	0.009	0.014	0.0165	0
Unclassified	0.676	0.5756	0.3333	0.4631	0.9554	0.4135	0.7736	0.9361

5.3 Intestinal microbiota composition at species level

Species	Control-72	Control-80	Control-20	Control-67	Model-36	Model-61	Model-97	Model-43
Lactobacillus_murinus	15.6342	15.7459	22.3661	12.3928	10.6161	31.9317	26.6171	23.5928
Helicobacter_ganmani	2.3103	1.1905	1.1601	0.7202	3.6791	12.8004	2.8368	13.7156
Akkermansia_muciniphila	1.9222	1.9141	1.6705	3.6565	1.432	0.3903	0.6627	0.5005
Bacteroides_acidifaciens	1.4215	0.718	1.3589	0.3544	0.3512	0.3682	0.3639	0.8687
Bacteroides_sartorii	1.5527	0.3534	0.2652	0.1848	0.4405	0.454	0.1571	0.3528
Faecalibaculum_rodentium	0.0509	1.2574	0.1171	0.0838	0.0155	0.0111	0.362	0.0265
Bacteroides_caecimuris	0.8647	0.2232	0.1436	0.1048	0.1358	0.1716	0.0575	0.0904
Clostridiales_bacterium_CIEAF_020	0.1339	0.0502	0.0199	0.0114	0.0272	0.0249	0.2548	0.0772
Desulfovibrio_fairfieldensis	0.3935	0.3199	0.0862	0.3277	0.1028	0.1052	0.0479	0.097
Helicobacter_hepaticus	0.1874	0.0446	0.1436	0.0248	0.0912	0.1772	0.069	0.1896
Other	0.7793	0.7385	0.3199	0.657	0.6285	0.9771	0.6967	0.4868
Unclassified	74.7496	77.444	72.3485	81.4812	82.4798	52.5882	67.8739	60.0017
Species	AS-L-2	AS-L-38	AS-L-100	AS-L-289	AS-H-1	AS-H-6	AS-H-68	AS-H-71
Lactobacillus_murinus	36.7968	52.7883	43.4898	32.8407	26.77	26.8745	19.394	26.9173
Helicobacter_ganmani	5.8591	2.0869	2.15	9.9637	8.7776	6.2813	5.6289	7.6071
Akkermansia_muciniphila	1.1572	0.3481	0.2182	1.1843	0.9607	2.0891	0.3365	0.5443
Bacteroides_acidifaciens	0.4162	0.2309	1.8151	0.4098	0.2689	0.0974	0.7443	0.1897
Bacteroides_sartorii	0.0426	0.0741	0.3113	0.1759	0.1577	0.0576	0.4974	0.1244
Faecalibaculum_rodentium	0.1259	0.0172	0.0102	0.0258	0.0663	0.2127	0.0841	0.0715
Bacteroides_caecimuris	0.0711	0.0379	0.1235	0.1678	0.0896	0.0735	0.0841	0.0933
Clostridiales_bacterium_CIEAF_020	0.0386	0.1017	1.2924	0.0307	0.0574	0.0656	0.0677	0.0373
Desulfovibrio_fairfieldensis	0.0995	0.1534	0.0846	0.0936	0.1362	0.0298	0.0421	0.0249
Helicobacter_hepaticus	0.1015	0.112	0.0913	0.234	0.1075	0.1411	0.3658	0.0529

Other	0.2801	0.8011	0.5958	0.4984	0.5399	0.7001	0.4701	0.3141
Unclassified	55.0115	43.2481	49.8182	54.3752	62.0684	63.3777	72.2853	64.0231

Domain	Phylum	Class	Order	Family	Genus
Bacteria	Firmicutes	Bacilli	Lactobacillales	Lactobacillaceae	Lactobacillus
Bacteria	Campilobacterota	Campylobacteria	Campylobacterales	Helicobacteraceae	Helicobacter
Bacteria	Verrucomicrobiota	Verrucomicrobiae	Verrucomicrobiales	Akkermansiaceae	Akkermansia
Bacteria	Bacteroidota	Bacteroidia	Bacteroidales	Bacteroidaceae	Bacteroides
Bacteria	Bacteroidota	Bacteroidia	Bacteroidales	Bacteroidaceae	Bacteroides
Bacteria	Firmicutes	Bacilli	Erysipelotrichales	Erysipelotrichaceae	Faecalibaculum
Bacteria	Bacteroidota	Bacteroidia	Bacteroidales	Bacteroidaceae	Bacteroides
Bacteria	Firmicutes	Clostridia	Lachnospirales	Lachnospiraceae	Lachnospiraceae_NK4A136_group
Bacteria	Desulfobacterota	Desulfovibrionia	Desulfovibrionales	Desulfovibrionaceae	Desulfovibrio
Bacteria	Campilobacterota	Campylobacteria	Campylobacterales	Helicobacteraceae	Helicobacter

5.4 Intestinal microbiota composition of Mucispirillum_schaedleri at genus level

	NO.	Mucispirillum_schaedleri	Average
Control	20	0.0088	
	67	0.0305	0.0319
	72	0.0696	
	80	0.0186	
Model	36	0.2445	
	61	0.454	0.2789
	43	0.1316	
	97	0.2854	
AS-L	2	0.0102	
	38	0.0241	0.0509
	100	0.0529	
	289	0.1162	
AS-H	1	0.1685	
	6	0.0398	
	68	0.0622	0.0772
	71	0.0684	

5.5 Good's coverage

index	sobs	shannon	simpson	chao	ace	goods_coverage	pielou	pd
Control-72	986	6.666201	0.964701	1102.683	1145.942	0.994217	0.670277	108.2574
Control-80	1172	6.392307	0.958453	1269.674	1323.177	0.995815	0.627019	130.011
Control-20	1015	6.035551	0.934132	1128.671	1170.376	0.995161	0.604325	111.8312
Control-67	1122	6.140538	0.958006	1263.143	1294.102	0.995275	0.606062	123.1611
Model-36	968	5.975265	0.95661	1093.158	1132.958	0.995692	0.602414	111.9063
Model-61	757	5.064979	0.874237	914.9333	934.9131	0.99427	0.52958	87.34998
Model-97	998	5.545084	0.911393	1111.046	1154.528	0.995748	0.556574	124.8702
Model-43	994	5.646766	0.916901	1129.819	1168.095	0.994863	0.567109	110.9128
AS-L-2	874	5.117472	0.854051	987.4606	1015.383	0.996061	0.523715	94.99949
AS-L-38	790	4.050006	0.714396	890.0621	926.6657	0.996898	0.420749	90.92264
AS-L-100	703	4.670447	0.80401	812.5938	841.649	0.997158	0.493842	80.37051
AS-L-289	786	5.096737	0.874144	907.25	951.3576	0.996854	0.529895	89.64967
AS-H-1	847	5.193724	0.904724	984.6707	1023.73	0.996182	0.533992	95.22357
AS-H-6	708	5.051301	0.897848	874.6911	901.6734	0.995965	0.533535	86.49226
AS-H-68	944	5.883212	0.942359	1060.777	1105.739	0.996068	0.595308	107.6667
AS-H-71	697	5.165332	0.902346	840.1655	878.0688	0.99378	0.546884	79.97185
